# North of England Women’s Diet and ActivitY - After Breast Cancer (NEWDAY-ABC) intervention in women diagnosed with early oestrogen-positive, HER2-negative breast cancer: a randomised controlled feasibility study

**DOI:** 10.1186/s40814-025-01689-3

**Published:** 2025-08-07

**Authors:** C. Wilson, K. Pickering, S. Wane, J. Cohen, C. Huang, M. Northgraves, H. Crank, A. Anderson, H. Cain, R. Copeland, J. Gray, J. Hargreaves, R. J. Q. McNally, J. M. Saxton

**Affiliations:** 1 The Christie NHS Foundation Trust, Manchester, UK; 2 Advanced Wellbeing Research Centre, Sheffield Hallam University, Old Hall Rd, Olympic Legacy ParkSheffield, S9 3TU UK; 3 Department of Sport, Exercise & Rehabilitation, Northumbria University, City Campus, Newcastle Upon Tyne, NE1 8 UK; 4 York Medical School, Hull Heath Trials Unit, University of Hull, Cottingham Road, HullHull, HU6 7RX UK; 5 Academy of Sport and Physical Activity, Sheffield Hallam University, Sheffield, S10 2BP UK; 6 Division of Population Health & Genomics, Centre for Research Into Cancer Prevention and Screening, Ninewells Hospital and Medical School, University of Dundee, Dundee, DD1 9SY UK; 7 Dept. of Breast Surgery, Newcastle Upon Tyne Hospitals NHS Foundation Trust, Queen Victoria Road, Newcastle Upon Tyne, NE1 4LP UK; 8 Department of Nursing, Northumbria University, Midwifery & HealthCoach Lane Campus, Newcastle Upon Tyne, NE7 7XA UK; 9 Carnegie School of Sport, Leeds Beckett University, 9 Fairfax Hall, Headingley Campus, Leeds, LS6 3QS UK; 10 Population Health Sciences Institute, Newcastle University, Newcastle University Centre for Cancer, Newcastle Upon Tyne, NE1 7RU UK; 11 School of Sport, Exercise & Rehabilitation Sciences, University of Hull, Cottingham Road, Hull, HU6 7RX UK

**Keywords:** Breast cancer, Diet, Physical activity, Weight management

## Abstract

**Background:**

Excess body weight is associated with higher breast cancer mortality rate. This study assessed the feasibility of a co-designed weight loss intervention (NEWDAY-ABC) versus standard care in early-stage oestrogen receptor-positive, human epidermal growth factor receptor 2-negative breast cancer patients.

**Methods:**

This was a two-arm, parallel group, randomised controlled feasibility study. Twenty-one ER + ve, HER2-ve stages I–III breast cancer patients, within 3 years of completing primary treatment (excluding endocrine therapy), were recruited from two UK National Health Service Breast Care Units and randomised (2:1) to intervention plus standard care or standard care alone. The intervention was co-designed with patients and comprised small group-based Support & Skills workshops delivered remotely via teleconference by trained lifestyle advisors and dieticians. Feasibility outcomes included recruitment rate, data quality, intervention acceptability and adherence. Exploratory clinical outcomes included weight loss, anthropometric measures, dietary change, physical activity and patient-reported outcomes.

**Results:**

Twenty-one women consented to the study, and 1 withdrew prior to randomisation, leaving 13 in the intervention group and 7 standard care controls, with 11 participants being followed up for 6 months. The overall attendance rate for intervention sessions was 79.6% (74/93 sessions completed). Body weight (candidate primary outcome for a fully powered randomised controlled trial) was reduced in the intervention group by 3.3 kg from baseline to 6 months, versus a 1.1 kg loss of body weight in the standard care control group. Furthermore, the European Organisation for Research and Treatment of Cancer Quality of Life Questionnaire-Core 30 (EORTC-QLQ30) breast module symptom scale scores for breast and arm symptoms improved in the intervention arm only, accompanied by positive changes in physical activity and dietary behaviours.

**Conclusion:**

The NEWDAY-ABC intervention is feasible and showed preliminary evidence of efficacy in terms of weight loss and other important health outcomes in women with early-stage breast cancer. The clinical and cost-effectiveness of the intervention versus standard care now needs to be robustly evaluated via an adequately powered clinical trial.

**Trial registration number:**

ISRCTN15088551, registered 3 February 2020.

**Supplementary Information:**

The online version contains supplementary material available at 10.1186/s40814-025-01689-3.

## Key messages regarding feasibility

- This study aimed to address uncertainties relating to the design and conduct of a definitive, phase III, randomised controlled trial including the following: (i) recruitment rate, (ii) delivery and adherence to the intervention and (iii) feasibility of collecting high-quality data on candidate health outcomes for a definitive trial.
- The study demonstrates the feasibility of implementation and provides preliminary evidence of efficacy, in terms of weight loss at 6 months and improved health-related quality of life.
- Our findings are limited to breast cancer patients from two recruitment sites in the North of England, and there are recognised cultural, economic and social differences between different regions. There is therefore a need for an adequately powered multicentre randomised controlled trial to test the effectiveness of this co-designed intervention in a broader population of early-stage breast cancer patients.

## Background

Primary treatment for early breast cancer involves surgery and multi-modality anticancer therapies which are associated with increased weight gain due to altered metabolism, changes in food intake and decreased activity levels [1]. Two-thirds of breast cancer patients are overweight/obese at diagnosis [2, 3], and inverse associations between body mass index (BMI) and survival outcomes have been reported. For example, a systematic literature review and meta-analysis by Chan et al. (2014) showed that a BMI above the normal range, measured 12 months before or after a breast cancer diagnosis, adversely impacts breast cancer specific survival, and women with obesity have a 33% increased risk of breast cancer-related mortality [4]. Studies that have evaluated associations between BMI and survival in different subtypes of breast cancer show that the most consistent evidence for the adverse impact of excess body weight is found for the commonest type of breast cancer, i.e. oestrogen receptor-positive, human epidermal growth factor receptor 2-negative (ER + ve HER2-ve) disease [5–7]. In the United Kingdom (UK), an analysis by the Oxford Early Breast Cancer Trialist Collaborative Group showed in the 60,000 women with ER + ve disease that higher BMI was associated with greater breast cancer mortality in pre-/perimenopausal women [8].

Interventions designed to support breast cancer patients in adopting healthy lifestyle behaviours (e.g. physical activity and healthy dietary choices) have much potential to attenuate the negative impact of raised BMI on breast cancer survival. Accordingly, an observational study of postmenopausal nurses in the United States of America (USA) with stages I–III breast cancer showed that the 10-year survival rate for women who engaged in nine metabolic equivalent of task (MET) hours/week of physical activity (3 MET hours = 1 h of brisk walking) was 92%, versus 86% in the < 3 MET hours/week group [9]. Additionally, a meta-analysis of 22 exercise studies (123,574 patients) showed a significantly lower breast cancer-related death (hazard ratio [HR]: 0.73, 95% confidence interval [CI] 0.54–0.98, *p* < 0.05) for patients reporting a high lifetime physical activity level compared to those with low physical activity [10]. Regarding dietary behaviour change, two randomised controlled trials in the USA evaluated a dietary reduction in fat after early breast cancer diagnosis (WINS [11] and WHEL [12]). The WINS study reported weight loss in the intervention group and a HR for recurrence in favour of the intervention vs control 0.76 (95 *CI* 0.6–0.98 *p* = 0.034). In contrast, the WHEL study reported no weight loss and with no difference in recurrent breast cancer or mortality between the intervention and control groups.

These results suggest that weight loss could be key for improving survival outcomes after breast cancer, and lifestyle interventions with both dietary and physical activity components are likely to have most impact. In this respect, studies show that interventions involving both dietary change and physical activity can elicit weight loss in the range of 5–10% and reduce serum levels of surrogate markers of breast cancer risk in breast cancer survivors and postmenopausal women with overweight [13–17]. The USA ENERGY trial evaluated such an intervention after ER + ve breast cancer and showed that age was strongly associated with weight loss (younger women losing less weight) [18], and the USA LISA trial showed that the efficacy of a lifestyle intervention on weight loss diminishes over time after cessation of intervention [19]. These trials suggest that a one-size-fits-all strategy for weight management may be insufficient for eliciting effective and long-lasting weight change in all women, and bespoke interventions addressing needs of specific populations of breast cancer survivors are required.

Through an initial qualitative, co-design phase of this project, we developed the North of England Women’s Diet and ActivitY After Breast Cancer (NEWDAY-ABC) lifestyle intervention for women with overweight after surgical treatment for early ER + ve, HER2-ve breast cancer [20, 21]. The primary aim of this study was to assess the feasibility and explore preliminary evidence of efficacy, to inform a large-scale randomised controlled trial of the intervention compared to standard care.

## Methods

### Study design

This was a two-arm, parallel group, randomised controlled feasibility study, to test the feasibility of the lifestyle intervention in ER + ve, HER2-ve breast cancer who have completed treatment with curative intent. Relevant clinical data and patient-reported outcome measures (PROMs) were collected to assess for signals of efficacy. The co-design methods have been published elsewhere [21]. Originally, the study was intended as an internal pilot within a randomised controlled trial; however, due to the challenges and research delays associated with the COVID-19 pandemic, it primarily assessed feasibility aspects under constrained circumstances. The study was sponsored by Northumbria University and was carried out in accordance with the Code of Ethics of the World Medical Association (Declaration of Helsinki) for experiments involving humans. Approval was granted by the North East NHS Research Ethics Committee (19-NE-0358) on the 7th January 2020, with the change in design to an external pilot/feasibility study approved on 11th December 2020. ISRCTN registration number is 15,088,551.

### Study population

Inclusion criteria are as follows:

- Within 3 years of completion of surgery/radiotherapy/chemotherapy for stages I–III ER + ve HER2-ve disease
- Overweight/obese (BMI ≥ 25 or > 30 kg/m^2^) and/or waist circumference > 88 cm
- Willing/able to attend group-based weight management workshops

Exclusion criteria are as follows:

- Metastatic/inoperable or active locoregional disease
- BMI < 25 kg/m.^2^
- Following alternative/complementary diets or taking high-dose antioxidant supplements for ≥ 3 months
- Severe physical/psychiatric impairments or severe comorbid diseases (e.g. arthritis/multiple sclerosis)
- Uncontrolled type 2 diabetes mellitus or cardiovascular disease
- Severe osteoporosis
- No telephone contact
- Unable to consent
- Enrolled on another weight loss trial
- Expecting to have surgery during the study
- Unable to speak/read English

### Participant recruitment

**Fig. 1:**
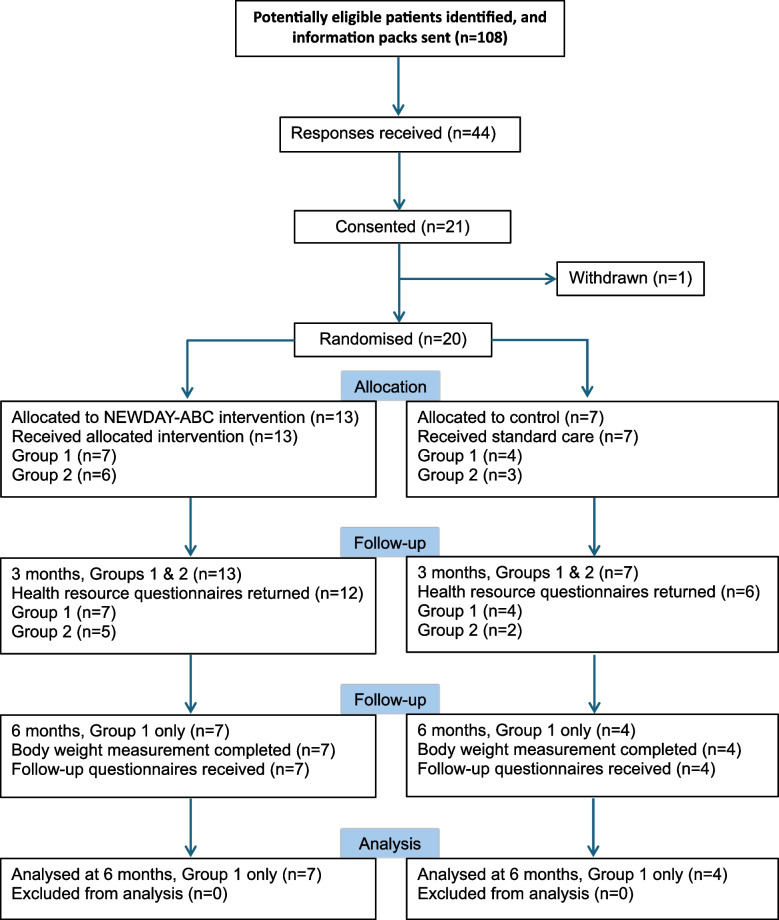
Consort flow chart

Site set-up was on schedule at three National Health Service (NHS) sites (Sheffield Teaching Hospitals NHS Trust, Gateshead Health NHS Trust and Northumbria Healthcare NHS Trust), but opening was delayed by 9 months due to non-COVID-19-related research being de-prioritised in the NHS. Due to this delay and funding being time restricted, recruitment time was limited to three calendar months (1 February 2021 to 30 April 2021), with intervention delivery and 6-month follow-up assessments needing to be completed by October 2021. Although the limited time window for recruitment impeded the opportunity to approach eligible patients about the study, it is possible that the COVID-19 pandemic also led to increased availability and willingness to participate due to fewer competing activities. Patients were recruited from three NHS Trusts in the UK (Sheffield Teaching Hospitals NHS Trust, Gateshead Health NHS Trust and Northumbria Healthcare NHS Trust). Recruitment was via postal invite from hospital datasets, direct approach in breast surgical/oncology clinics, and advertisement in GP surgeries. Participants were recruited and randomised as two distinct cohorts (Group 1: *n* = 11; Group 2; *n* = 9) to facilitate the formation of small groups for the Support & Skills workshops which was key component of the intervention (Fig. 1).

### Screening and consent

Interested patients were telephoned by the research team who screened for eligibility. Screening for BMI and waist circumference was based on self-reported measurements. Remote postal informed consent or e-consent was obtained from participants for all aspects of the study, and participants were free to withdraw at any time.

### Randomisation

The Hull Health Trials Unit (HHTU) developed a web-based database and randomisation system using a secure, cloud-based platform for building and managing research databases (REDCap Cloud, CA, USA). A HHTU statistician, independent from the study team, prepared the randomisation schedule. Participants were randomised (2:1) to intervention or standard care/control using random permuted blocks. Randomisation was stratified to balance the potentially confounding variables of site, chemotherapy and type of endocrine treatment, though we acknowledge that given the number of strata resulting from the specified stratification variables, along with lower-than-anticipated recruitment, a randomisation by minimisation approach may have been a more appropriate method. The nature of the intervention meant that the blinding of participants was not possible. The 6-month outcome assessor was blinded to group allocation.

### Control group

The control group received standard care alone which normally comprises a 5–10-year course of adjuvant endocrine therapy in women with ER + ve tumours with face-to-face, virtual or self-reporting NHS secondary care follow-up. Control participants received the printed intervention materials at the end of study.

### Intervention

**Fig. 2:**
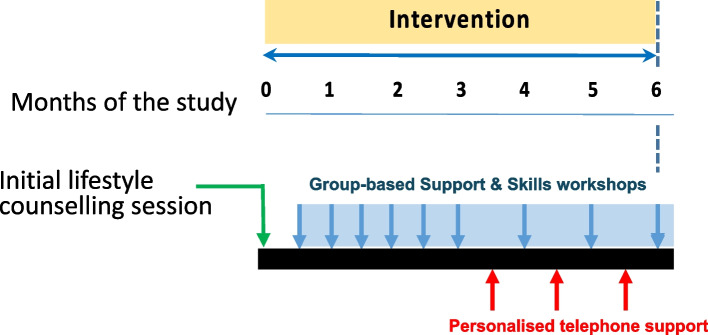
Intervention overview

Details of the intervention co-design stages have been published previously [20, 21]. The intervention was codesigned as a 6-month programme of group-based support, followed by 6–12 months of maintenance support, with workshops delivered remotely via teleconference by trained lifestyle advisors (trained to Register of Exercise Professionals Level 4 in Exercise and Cancer) and dieticians. However, the challenges imposed by the COVID-19 pandemic meant that only the first 6 months of the group-based support programme could be piloted and assessed for feasibility (Fig. 2), and not all participants had reached the 6-month follow-up when the study was closed. Furthermore, the intervention had to be delivered entirely remotely with patients self-reporting weight, height, waist and hip circumference (under the guidance of researchers K. P./S. W.). Intervention workshops were complemented by telephone/email support, and participants had access to support from their peers and a lifestyle advisor via a bespoke web platform. Workshops covered the following topics: portion sizes, confidence, mood, lifestyle, alcohol and drinks, eating and moving as a family [21].

### Outcomes

As a feasibility study, the main aim was to address uncertainty relating to the design and conduct of a definitive, phase III, randomised controlled trial, as follows:

- Recruitment rate (feasibility criterion: > 35% eligible patients recruited)
- Delivery and adherence to intervention (feasibility criterion: ≥ 60% attendance at workshops)
- Data quality and completeness for candidate primary and secondary outcomes for a definitive trial, including NHS resource use data for cost-effectiveness analysis (feasibility criterion: > 90% data collection).

Clinical outcomes were included as a means of identifying early indicative evidence of efficacy in key candidate outcomes for a phase III trial, with data collected at baseline and 6-month post-randomisation. Potential co-primary outcomes for a definitive randomised controlled trial were as follows:

- Change in body weight at 6 months
- Patient-reported health-related quality of life (HRQoL), assessed by the European Organisation for Research and Treatment of Cancer Quality of Life Questionnaire-Core 30 (EORTC QLQ-C30) [22] and EORTC Breast Module (BR-23) [23].

Proposed secondary outcomes for a definitive trial were also collected and included the following:

- Anthropometric: Height, waist and hip girths, and BMI
- Physical activity: Modified Godin Leisure-Time Exercise Questionnaire [24] and 7-day free-living physical activity using accelerometry
- PROMs: Fatigue assessed using the Functional Assessment of Chronic Illness Therapy (FACIT) Fatigue Scale [25], body image using an established 10-item instrument [26, 27] and fear of cancer recurrence (FCR) assessed using FCR7 [28, 29]
- Diet: Eating behaviours using a bespoke questionnaire for diet and alcohol consumption using the Alcohol Use Disorders Identification Test-Consumption (AUDIT-C) [30]
- NHS resource use patient diary and European Quality of Life 5 Dimensions 5-Level Version (EQ-5D-5L) [31]

### Data collection

PROMs were completed as paper or online questionnaires, depending on patient preference, and accelerometers were issued, with the intention of recording 7-day free-living physical activity. The 6-month assessments were conducted by a researcher blinded to group allocation. A member of the site clinical research team reviewed the medical records for adverse events (AEs), the reporting period for which ended at the patient’s final follow-up contact, and with only AEs related to the study being recorded. Data collection, processing and storage in REDCap Cloud were handled in a way that complies with the General Data Protection Regulations.

### Sample size and statistical methods

As a feasibility trial, a formal sample size calculation was not required. We aimed to recruit 30 patients in accordance with published recommendations [32], to assess the feasibility parameters of the study as follows: (i) delivery of the intervention as planned, including 2 ‘closed-group’ intervention cohorts of patients in each of the two centres, (ii) ability to recruit women at the rate necessary to complete a fully powered randomised controlled trial within 30 months, (iii) 60% adherence to workshops (intervention group only) and (iv) 80% retention of participants on the trial. The flow of individual participants through each stage of the trial was reported, in accordance with the Consolidated Standards of Reporting Trials (CONSORT) 2010 statement extension for pilot and feasibility trials [33]. Descriptive statistics (mean, standard deviation [SD] and range for continuous variable; count and % for categorical variable) were used to summarise data at baseline and 6-month follow-up, together with the group differences at 6 months (95% CI), adjusting for baseline scores to ensure that natural baseline variation was accounted for. All analyses were conducted using SPSS version 27 (SPSS Inc., Chicago, IL, USA).

## Results

The consort diagram for the flow of participants through the feasibility study is displayed in Fig. 1. Only the first cohort randomised (Group 1: *n* = 11) reached the 6-month follow-up timepoint before the study was closed, representing 55% of the randomised study population. However, 3-month resource use data were collected for both cohorts of participants (Group 1: *n* = 11 and Group 2: *n* = 9).

### Baseline characteristics

Participants had a mean (SD) age of 54.7 (8.8) years, were predominately white (95%), married (65%) and with children (80%). All participants were receiving hormone therapy and had undergone surgery as part of their breast cancer treatment, with 75% having received chemotherapy. The overall balance of baseline demographics indicated the success of randomisation between intervention and control groups (Table 1).

**Table 1:** Baseline characteristics

Characteristics	Intervention group *n* = 13	Control group *n* = 7	Overall *n* = 20
**Age (year)**, mean (SD) [range]	55.9 (10.0) [36–69]	52.3 (6.0) [40–59]	54.7 (8.8) [36–69]
**Ethnicity, *n* (%)**			
White British	13 (100)	6 (85.)	19 (95)
Black or Black British	0 (0)	1 (14.3)	1 (5)
**Education, *n* (%)**			
Secondary school	2 (15.4)	1 (14.)	3 (15)
Higher education/technical/vocational qualification	7 (53.8)	5 (71.5)	12 (6)
University degree/postgraduate degree	4 (30.8)	1 (14.3)	5 (25)
**Marital status, *n* (%)**			
De facto	1 (7.7)	1 (14.3)	2 (10)
Divorced/separated	0 (0)	1 (14.3)	1 (5)
Married	11 (84.6)	2 (28.6)	13 (65)
Single	1 (7.7)	3 (42.9)	4 (20)
**Child, *n* (%)**			
Yes	10 (76.9)	6 (85.7)	16 (80)
No	3 (23.1)	1 (14.3)	4 (20)
**Body weight (kg)**, mean (SD) [range]	80.8 (9.4) [69.0–101.0]	82.0 (3.8) [78.9–88.5]	81.2 (7.9) [69.0–101.0]
**Height (m)**, mean (SD) [range]	1.65 (0.05) [1.57–1.73]	1.62 (0.06) [1.52–1.69]	1.64 (0.05) [1.52–1.73]
**BMI (kg/m**^**2**^**)**, mean (SD) [range]	29.7 (3.4) [25.5–37.6]	31.6 (3.0) [27.7–34.6]	30.3 (3.3) [25.5–37.6]
**Waist circumference (cm)**, mean (SD) [Range]	92.4 (10.1) [71.3–111.0]	95.5 (4.2) [91.0–102.5]	93.4 (8.5) [71.3–111.0]
**Hip circumference (cm)**, mean (SD) [range]	107.7 (8.4) [92.5–125.0]	108.6 (6.7) [99.0–118.0]	108.0 (7.7) [92.5–125.0]

### Feasibility outcomes

#### Recruitment rate

Information packs were sent to 108 potentially eligible patients, with 44 responses (40.7% response rate) being received, of which 32 patients (72.7%) expressed an interest in the study and were confirmed as being eligible. Of these, 21 women consented to the study representing a 66% recruitment rate of eligible patients. One patient withdrew prior to randomisation.

#### Intervention attendance and acceptability

**Fig. 3:**
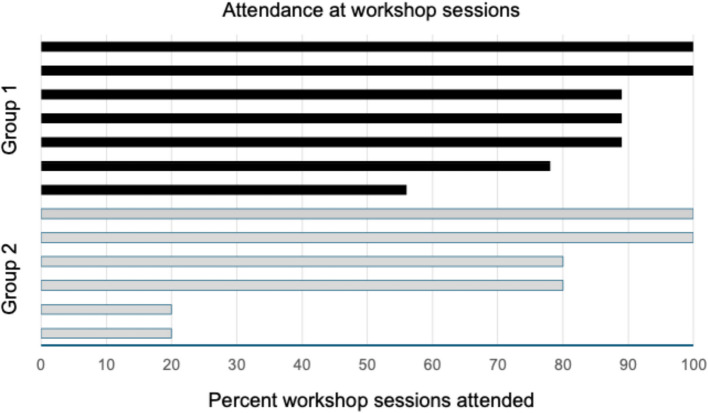
Participant attendance at Support & Skills workshops expressed as a percentage of scheduled sessions attended. Note that Group 1 had nine scheduled sessions and Group 2 had five scheduled sessions

All participants in the first cohort (Group 1: *n* = 7) completed the 6-month intervention and were offered nine Support & Skills workshops during this time period. The second cohort of participants (Group 2: *n* = 6) engaged in approximately 3 months of the intervention before study closure and were offered five Support & Skills workshops during this time-period (Fig. 3). The overall attendance rate was 79.6% (74/93 sessions), with 10 out of the 13 participants randomised to the intervention group (Group 1: *n* = 6; Group 2: *n* = 4) completing at least 75% of Support & Skills workshops. Individual attendance ranged from 56%–100% in group 1 to 20%–100% in Group 2.

#### Data quality

Data completeness for the candidate primary outcomes (body weight and EORTC QLQ-30 and BR23), secondary anthropometric outcomes (BMI, waist circumference and hip circumference) and PROMs (AUDIT-C, Body Image Scale, FCR7, FACIT-Fatigue and EQ-5D-5L) was 100% for baseline and follow-up in the first cohort of participants (Group 1: *n* = 11), and the self-reported Modified Godin Leisure-Time Exercise Questionnaire was fully completed by 10 out of 11 participants (91%). NHS resource use data were collected from 18 out of 20 participants (90% Groups 1 & 2) at 3 months and 11 out of 11 (100%) at 6 months (Group 1 only).

#### Safety

There were no AEs during the study.

#### Body weight and anthropometric outcomes

All seven intervention participants reaching the 6-month follow-up experienced a reduction in body weight, resulting in a group mean reduction (Table 2). Three of the four participants in the control group also had a small reduction in body weight, resulting in a smaller group reduction of 1.3% (83.4 to 82.3 kg). The adjusted mean difference in body weight between the two groups was − 2.5 kg (95% *CI*: − 5.9, 0.8) in favour of the intervention group. A similar pattern favouring the intervention was seen for the secondary outcomes of BMI (mean difference: − 2.1 kg/m^2^; 95% *CI*: − 4.1, − 0.1), waist circumference (− 0.7 cm; 95% *CI*: − 4.6, 3.3) and hip circumference (− 5.0 cm, 95% *CI*: − 7,4, − 2.6).

**Table 2:** Baseline and 6-month body weight and anthropometric data for intervention and control group participants

	**Intervention group** Mean (SD) [range] *n* = 7		**Control group** Mean (SD) [range] *n* = 4		**Mean difference (95% *CI*) at 6 months adjusting for baseline score**
	Baseline	6 months	Baseline	6 months	
Body weight (kg)	79.5 (5.4) [71.0–84.8]	76.2 (5.1) [68.0–82.1]	83.4 (4.0) [78.9–88.5]	82.3 (4.4) [76.2–86.6]	− 2.5 (− 5.9, 0.8)
BMI (kg/m^2^)	28.4 (1.6) [25.5–30.4]	27.3 (1.5) [24.4–28.8]	33.4 (1.2) [32.1–34.6]	33.0 (0.6) [32.3–33.8]	− 2.1 (− 4.1, − 0.1)
Waist circumference (cm)	90.4 (5.3) [85.0–98.0]	88.9 (5.9) [82.6–97.0]	97.1 (5.0) [92.7–102.5]	97.2 (7.2) [89.2–106.0]	− 0.7 (− 4.6, 3.3)
Hip circumference (cm)	107.7 (4.9) [101.0–116.0]	104.4 (4.7) [99.1–112.0]	111.5 (6.6) [103.0–118.0]	112.6 (5.0) [107.0–117.9]	− 5.0 (− 7,4, − 2.6)

#### Patient-reported outcome measures (PROMs)

For the co-candidate co-primary outcome of EORTC-QLQ30/EORTC BR-23, the intervention group showed a numerically greater improvement in EORTC BR-23 breast module functional scores compared to the control group (supplementary table). Symptom scales showed that systemic therapy side effects increased from baseline to the 6-month follow-up in both groups; however, breast and arm symptoms were improved in the intervention group only. The fear of cancer recurrence score declined to a greater extent in the intervention vs control group (− 3.0 vs − 0.3, respectively), and the FACIT fatigue scale showed a numerical improvement in fatigue symptoms amongst intervention participants at the 6-month follow-up. An important caveat is that the control group consistently scored worse on several PROMs at baseline (supplementary table) while also having a higher baseline BMI (Table 2).

The self-reported Modified Godin Leisure-Time Exercise Questionnaire showed a pattern for either increased number of sessions or intensity of physical intensity in six of the seven participants (86%) in the intervention group compared with two of the four participants (50%) in the control group. Due to challenges with the timely collection of accelerometers during the COVID-19 period and some technical issues, accelerometer data were not analysed. There were positive signals of changes in diet and eating behaviours at the 6-month follow-up. Compared to baseline, the number of snacks per week was reduced in five of the seven participants in the intervention group. In contrast, only one of the four participants in the control group reported reduced snack consumption. Fruit portions per day increased in three of the seven participants in the intervention group compared with one of the four participants in the control group. Vegetable portions per day (71% versus 0%) and sugary drink consumption (57% versus 50%) were also greater in the intervention groups versus controls, respectively, at the 6-month follow-up, and alcohol consumption increased in both groups.

## Discussion

The results of this feasibility study suggest that it is feasible to recruit breast cancer patients and deliver this co-designed intervention in a UK setting, and that it has the potential to provide effective weight management support for women recovering from primary ER + ve breast cancer treatment. Patient recruitment to the feasibility study and delivery of the intervention was challenged by the COVID-19 pandemic. Because of this, recruitment had to be completed within three calendar months. Furthermore, this was a time of significant fear and anxiety amongst cancer patients who were being told to shield, thereby impacting their willingness to engage with clinical trials. However, the response rate to our invitation letters, and the resulting conversion to consent rate of 66% of participants showing an interest in the study, strongly suggests that recruitment would be feasible in a larger trial. Furthermore, attendance at the remotely delivered workshops for women randomised to intervention was ~ 80%, and candidate primary outcomes for a fully powered trial had a 100% completion rate in participants that were followed-up for 6 months.

Preliminary indications of intervention efficacy are evident in our weight loss data. Participants randomised to the intervention showed a superior weight loss, accompanied by improvements in dietary behaviours, self-reported physical activity and PROMs. Despite the challenges of the COVID-19 pandemic, 4.2% weight loss was observed for the intervention group at the 6-month follow-up, which is only marginally less than that previously reported for ER + ve breast cancer patients in prominent North American trials. For example, the ENERGY trial [18] reported weight loss of 5.9% at 6 months in the intervention group, and the LISA trial [19] reported 5.3% weight loss at 6 months. Our intervention was adapted for remote delivery via Zoom, and this may have attenuated the amount of weight loss achieved due to lack of the face-to-face support options for patients. In this respect, the LEAN study [34] compared in-person counselling with telephone counselling in breast cancer survivors with overweight and reported more weight loss in the in-person group (6.4% and 5.4%, respectively). Most recently, in the largest randomise controlled trial to date, the ongoing BWEL study (USA and Canada) has recruited women with a BMI > 27 to a 2-year telephone intervention versus control and has reported interim weight loss of 4.8% (± 7.9) at 12 months in the intervention group versus 0.8% (± 6.4) of weight gain in the controls [35]. A key difference between these North American trials and the NEWDAY-ABC intervention is the high level of patient involvement in design of the latter. Using co-design methods to address common barriers to weight management behaviours and help to facilitate positive health behaviour change in this context, we aim to demonstrate the impact of NEWDAY-ABC on longer-term weight loss and weight loss maintenance in early breast cancer patients with overweight via an appropriately powered clinical trial.

Living with excess body weight has negative consequences for patients’ HRQoL, including low self-esteem, body image concerns and fear of recurrence [36–38]. PROMs data for the intervention group reassuringly showed an improvement in multiple HRQoL scores including body image, breast and arm symptoms, fear of cancer recurrence and sexual functioning. Whilst the feasibility study was not powered to detect significant changes in these outcomes, the signals of efficacy are reassuring for inclusion of these as important secondary endpoints in a future randomised controlled trial. However, the control group consistently scored worse at baseline on several PROMs, and this may have had some bearing on the results. This important caveat highlights the need for caution when interpreting signals of efficacy for the PROMs data reported herein.

From a dietary perspective, our study showed an increase in daily fruit and vegetable consumption and fewer weekly snacks in the intervention group. Similar alterations in dietary behaviours have been reported in other breast cancer lifestyle intervention studies; for example, the LEAN study reported a greater increase in fruit and vegetable consumption and a decrease in fat intake in the intervention group versus controls [39]. If maintained, the benefits of such dietary change could not only impact future cancer recurrence risk but could also reduce the risk of future noncommunicable comorbidities associated with poor health behaviours. However, there was evidence of an increased consumption of alcohol and sugary drinks in both groups, suggesting these dietary behaviours need to be more strongly targeted in a fully-powered trial. Aside from the positive dietary changes, our results indicated an increase in all intensities of physical activity in the intervention group, despite shielding advise given to cancer patients during the COVID-19 pandemic and closure of community exercise facilities. A physically active lifestyle after primary ER + ve breast cancer treatment improves quality of life and physical functioning [38, 40] and has an important role in long-term weight loss maintenance [41, 42].

Definitive intervention trials are needed to corroborate observational evidence and confirm the impact of weight loss on clinical endpoints. Three large-scale randomised controlled trials in Europe and the USA are currently evaluating this. The German SUCCESS-C study [43] has recruited 2292 women with overweight who have been diagnosed with *T*_1–4_, *N*_0–3_ and HER2-ve disease and randomised them to a 2-year telephone intervention vs control, with the primary outcome being disease-free survival. The Italian DIANA-5 study [44] has randomised 1208 women with early stage, predominantly ER + ve breast cancer, at high risk of recurrence to a lifestyle intervention and aims to evaluate the impact of the intervention on local/distant recurrence. Finally, the large-scale BWEL study [45] in the USA is a phase III trial evaluating a 2-year telephone lifestyle intervention vs control in HER2- stage II/III breast cancer patients with overweight. The trial is aiming to recruit 3136 patients and will evaluate invasive disease-free survival. These trials have much potential to define the link between weight loss and breast cancer-specific survival and provide definitive data on how much weight loss is associated with invasive disease-free survival. Furthermore, the Dutch OPTIMUM study [46] is evaluating optimal timing to promote a lifestyle intervention after breast cancer to gain long-term adherence and will also asses the optimal method for cancer health care professionals to promote these lifestyle interventions to reduce body weight, which will assist in timing of recruitment to future lifestyle intervention studies. UK-based interventions with proven preliminary evidence of efficacy (e.g. NEWDAY-ABC) will benefit from data generated by these international trials by enabling extrapolation of weight loss to long-term breast cancer outcomes. This will then provide a stronger rationale for a standardised pan-NHS approach to weight management support after a breast cancer diagnosis.

### Study limitations

Although the intervention elicited initial evidence of weight loss at 6 months, we do not have longer-term follow-up data beyond this time-point to know if the women would maintain weight loss or continue to lose weight during the maintenance support phase of the intervention. In the LISA trial [19], the 5% weight loss seen in the intervention arm at 6 and 12 months decreased to 3.3% at 24 months and disappeared by 84 months suggesting it is critical to maintain longer-term follow-up and support for these women, which is what the larger-scale NEWDAY-ABC trial would intend to do. COVID-19 prevented the collection of serum samples (for biomarker analysis) and meant that patients self-reported weight and other anthropometric measures under the guidance of researcher. This may have increased measurement error, compromising the validity of the weight loss data in this non-blinded, randomised intervention trial. The COVID-19 pandemic also meant that patients were unable to choose their preferred delivery style (virtual vs face to face). Finally, the intervention was piloted in a north of England breast cancer population, and there are recognised cultural, economic and social differences between the north and south of England which could result in the results not being applicable to a south of England breast cancer population.

## Conclusion

We have successfully piloted a co-designed lifestyle intervention leading to weight loss in ER + ve HER2-ve patients with overweight in the north of England, but there remains a need for a large, multicentre randomised controlled trial to evaluate if this co-designed intervention can significantly reduce weight in a broader geographical population. The candidate co-primary outcomes (body weight and HRQoL) and proposed secondary outcomes (including PROMs) were acceptable to participants and yielded promising preliminary evidence of the health benefits to be gained from the intervention. We were also able to collect a high level of NHS resource data from participants. This provides a rationale for the use of these primary and secondary outcomes in a future fully powered trial, as a means of evaluating the clinical and cost-effectiveness of NEWDAY-ABC and its suitability for national commissioning, in line with interventions that are already in place for patients with T2DM and coronary heart disease.

## Supplementary Information

Additional file 1: Supplementary table: Results.

## Data Availability

The datasets used and/or analysed during the current study available from the corresponding author on reasonable request.

## References

[1] McTiernan A. Weight, physical activity and breast cancer survival. Proc Nutr Soc. 2018;77(4):403–11. 29478430 10.1017/S0029665118000010

[2] Cecchini RS, Swain SM, Costantino JP, Rastogi P, Jeong JH, Anderson SJ, et al. Body mass index at diagnosis and breast cancer survival prognosis in clinical trial populations from NRG Oncology/NSABP B-30, B-31, B-34, and B-38. Cancer Epidemiol Biomarkers Prev. 2016;25(1):51–9. 26545405 10.1158/1055-9965.EPI-15-0334-T PMC4713289

[3] Anderson AS, Martin RM, Renehan AG, Cade J, Copson ER, Cross AJ, et al. Cancer survivorship, excess body fatness and weight-loss intervention-where are we in 2020? Br J Cancer. 2021;124(6):1057–65. 33235316 10.1038/s41416-020-01155-2 PMC7961062

[4] Chan DSM, Vieira AR, Aune D, Bandera EV, Greenwood DC, McTiernan A, et al. Body mass index and survival in women with breast cancer-systematic literature review and meta-analysis of 82 follow-up studies. Ann Oncol. 2014;25(10):1901–14. 24769692 10.1093/annonc/mdu042 PMC4176449

[5] Ewertz M, Gray KP, Regan MM, Ejlertsen B, Price KN, Thurlimann B, et al. Obesity and risk of recurrence or death after adjuvant endocrine therapy with letrozole or tamoxifen in the breast international group 1–98 trial. J Clin Oncol. 2012;30(32):3967–75. 23045588 10.1200/JCO.2011.40.8666 PMC3488270

[6] Sestak I, Distler W, Forbes JF, Dowsett M, Howell A, Cuzick J. Effect of body mass index on recurrences in tamoxifen and anastrozole treated women: an exploratory analysis from the ATAC trial. J Clin Oncol. 2010;28(21):3411–5. 20547990 10.1200/JCO.2009.27.2021

[7] Niraula S, Ocana A, Ennis M, Goodwin PJ. Body size and breast cancer prognosis in relation to hormone receptor and menopausal status: a meta-analysis. Breast Cancer Res Treat. 2012;134(2):769–81. 22562122 10.1007/s10549-012-2073-x

[8] Pan HGR. Effect of obesity in premenopausal ER+ early breast cancer: EBCTCG data on 80,000 patients in 70 trials. J Clin Oncol. 2014;32:5s, (suppl; abstr 503).

[9] Holmes MD, Chen WY, Feskanich D, Kroenke CH, Colditz GA. Physical activity and survival after breast cancer diagnosis. JAMA. 2005;293(20):2479–86. 15914748 10.1001/jama.293.20.2479

[10] Lahart IM, Metsios GS, Nevill AM, Carmichael AR. Physical activity, risk of death and recurrence in breast cancer survivors: a systematic review and meta-analysis of epidemiological studies. Acta Oncol. 2015;54(5):635–54. 25752971 10.3109/0284186X.2014.998275

[11] Hoy MK, Winters BL, Chlebowski RT, Papoutsakis C, Shapiro A, Lubin MP, et al. Implementing a low-fat eating plan in the women’s intervention nutrition study. J Am Diet Assoc. 2009;109(4):688–96. 19328264 10.1016/j.jada.2008.12.016 PMC5108363

[12] Pierce JP, Faerber S, Wright FA, Rock CL, Newman V, Flatt SW, et al. A randomized trial of the effect of a plant-based dietary pattern on additional breast cancer events and survival: the Women’s Healthy Eating and Living (WHEL) study. Control Clin Trials. 2002;23(6):728–56. 12505249 10.1016/s0197-2456(02)00241-6

[13] Pakiz B, Flatt SW, Bardwell WA, Rock CL, Mills PJ. Effects of a weight loss intervention on body mass, fitness, and inflammatory biomarkers in overweight or obese breast cancer survivors. Int J Behav Med. 2011;18(4):333–41. 21336679 10.1007/s12529-010-9079-8 PMC3212681

[14] Rock CL, Pande C, Flatt SW, Ying C, Pakiz B, Parker BA, et al. Favorable changes in serum estrogens and other biologic factors after weight loss in breast cancer survivors who are overweight or obese. Clin Breast Cancer. 2013;13(3):188–95. 23375717 10.1016/j.clbc.2012.12.002 PMC4153749

[15] Travier N, Buckland G, Vendrell JJ, Fernandez-Veledo S, Peiro I, Del Barco S, et al. Changes in metabolic risk, insulin resistance, leptin and adiponectin following a lifestyle intervention in overweight and obese breast cancer survivors. Eur J Cancer Care (Engl). 2018;27(4):e12861. 29869823 10.1111/ecc.12861

[16] Alfano CM, Imayama I, Neuhouser ML, Kiecolt-Glaser JK, Smith AW, Meeske K, et al. Fatigue, inflammation, and omega-3 and omega-6 fatty acid intake among breast cancer survivors. J Clin Oncol. 2012;30(12):1280–7. 22412148 10.1200/JCO.2011.36.4109 PMC3341143

[17] van Gemert WA, Schuit AJ, van der Palen J, May AM, Iestra JA, Wittink H, et al. Effect of weight loss, with or without exercise, on body composition and sex hormones in postmenopausal women: the SHAPE-2 trial. Breast Cancer Res. 2015;17:120. 26330303 10.1186/s13058-015-0633-9 PMC4557857

[18] Rock CL, Flatt SW, Byers TE, Colditz GA, Demark-Wahnefried W, Ganz PA, et al. Results of the Exercise and Nutrition to Enhance Recovery and Good Health for You (ENERGY) trial: a behavioral weight loss intervention in overweight or obese breast cancer survivors. J Clin Oncol. 2015;33(28):3169–76. 26282657 10.1200/JCO.2015.61.1095 PMC4582146

[19] Goodwin PJ, Segal RJ, Vallis M, Ligibel JA, Pond GR, Robidoux A, et al. The LISA randomized trial of a weight loss intervention in postmenopausal breast cancer. NPJ Breast Cancer. 2020;6:6. 32133391 10.1038/s41523-020-0149-z PMC7035359

[20] Saxton JM P, K., Wane, S., Crank, H., Anderson, A.S., Cain, H., Cohen, J., Copeland, R.J., Gray J., Hargreaves, J., McNally, R.J.Q., Wilson, C.: . The experiences and perceptions of breast cancer patients regarding weight management during and after treatment for oestrogen-receptor positive disease: a qualitative study. BMC Cancer. 2022. 10.1186/s12885-022-10238-.

[21] Saxton JM, Pickering K, Wane S, Humphreys H, Crank H, Anderson AS, et al. Co-designed weight management intervention for women recovering from oestrogen-receptor positive breast cancer. BMC Cancer. 2022;22(1):1202. 36418985 10.1186/s12885-022-10287-y PMC9682743

[22] Aaronson NK, Ahmedzai SA, Bergman B, Bulinger M, Cull A, Duez NJ, et al. The EORTC QLQ-C30 - a quality-of-life instrument for use in international clinical trials in oncology. J Natl Cancer Inst. 1993;85(5):365–76. 8433390 10.1093/jnci/85.5.365

[23] Sprangers MA, Groenvold M, Arraras JI, Franklin J, te Velde A, Muller M, et al. The European organization for research and treatment of cancer breast cancer-specific quality-of-life questionnaire module: first results from a three-country field study. J Clin Oncol. 1996;14(10):2756–68. 8874337 10.1200/JCO.1996.14.10.2756

[24] Amireault S, Godin G, Lacombe J, Sabiston CM. Validation of the Godin-shephard leisure-time physical activity questionnaire classification coding system using accelerometer assessment among breast cancer survivors. J Cancer Surviv. 2015;9(3):532–40. 25666749 10.1007/s11764-015-0430-6

[25] Yellen SB, Cella DF, Webster K, Blendowski C, Kaplan E. Measuring fatigue and other anemia-related symptoms with the Functional Assessment of Cancer Therapy (FACT) measurement system. J Pain Symptom Manage. 1997;13(2):63–74. 9095563 10.1016/s0885-3924(96)00274-6

[26] Hopwood P, Fletcher I, Lee A, Al GS. A body image scale for use with cancer patients. Eur J Cancer. 2001;37(2):189–97. 11166145 10.1016/s0959-8049(00)00353-1

[27] Baxter NN, Goodwin PJ, McLeod RS, Dion R, Devins G, Bombardier C. Reliability and validity of the body image after breast cancer questionnaire. Breast J. 2006;12(3):221–32. 16684320 10.1111/j.1075-122X.2006.00246.x

[28] Humphris GM, Watson E, Sharpe M, Ozakinci G. Unidimensional scales for fears of cancer recurrence and their psychometric properties: the FCR4 and FCR7. Health Qual Life Outcomes. 2018;16(1):30. 29471823 10.1186/s12955-018-0850-x PMC5822647

[29] Humphris G, Watson E, Sharpe M, Ozakinci G. Unidimensional scales for fears of cancer recurrence and their psychometric properties: the FCR4 and FCR7. 2018;16(1):30. 10.1186/s12955-018-0850-x PMC5822647 29471823

[30] Zhu J, Jiang X, Niu Z. Alcohol consumption and risk of breast and ovarian cancer: a Mendelian randomization study. Cancer Genet. 2020;245:35–41. 32585585 10.1016/j.cancergen.2020.06.001

[31] Herdman M, Gudex C, Lloyd A, Janssen M, Kind P, Parkin D, et al. Development and preliminary testing of the new five-level version of EQ-5D (EQ-5D-5L). Qual Life Res. 2011;20(10):1727–36. 21479777 10.1007/s11136-011-9903-x PMC3220807

[32] Lancaster GA, Dodd S, Williamson PR. Design and analysis of pilot studies: recommendations for good practice. J Eval Clin Pract. 2004;10(2):307–12. 15189396 10.1111/j..2002.384.doc.x

[33] Eldridge SM, Chan CL, Campbell MJ, Bond CM, Hopewell S, Thabane L, et al. CONSORT 2010 statement: extension to randomised pilot and feasibility trials. Pilot Feasibility Stud. 2016;2:64. 27965879 10.1186/s40814-016-0105-8 PMC5154046

[34] Harrigan M, Cartmel B, Loftfield E, Sanft T, Chagpar AB, Zhou Y, et al. Randomized trial comparing telephone versus in-person weight loss counseling on body composition and circulating biomarkers in women treated for breast cancer: the lifestyle, exercise, and nutrition (LEAN) study. J Clin Oncol. 2016;34(7):669–76. 26598750 10.1200/JCO.2015.61.6375 PMC4872022

[35] Ligibel JA, Ballman, K. V., McCall, L. M., Goodwin, P. J., Weiss, A., Delahanty, L., Alfano, C. M., Crane, T. E., Neuhouser, M. L., Spears, P., Hershman, D. L., Paskett, E. D., Hopkins, J. O., Bernstein, V., Stearns, V., White, J. R., Wadden, T., Winer, E. P., Partridge, A. H., Carey, L. A. 2023 ASCO Annual Meeting: effect of a telephone-based weight loss intervention (WLI) on weight at 12-months in women with early breast cancer: results from the Breast Cancer Weight Loss (BWEL) trial (abstract 12001). Journal of Clinical Oncology. 2023;41_Supplement 16.

[36] Mustian KM, Alfano CM, Heckler C, Kleckner AS, Kleckner IR, Leach CR, et al. Comparison of pharmaceutical, psychological, and exercise treatments for cancer-related fatigue: a meta-analysis. JAMA Oncol. 2017;3(7):961–8. 28253393 10.1001/jamaoncol.2016.6914 PMC5557289

[37] Juvet LK, Thune I, Elvsaas IKO, Fors EA, Lundgren S, Bertheussen G, et al. The effect of exercise on fatigue and physical functioning in breast cancer patients during and after treatment and at 6 months follow-up: a meta-analysis. Breast. 2017;33:166–77. 28415013 10.1016/j.breast.2017.04.003

[38] Zhang X, Li Y, Liu D. Effects of exercise on the quality of life in breast cancer patients: a systematic review of randomized controlled trials. Support Care Cancer. 2019;27(1):9–21. 30032399 10.1007/s00520-018-4363-2

[39] Anderson C, Harrigan M, George SM, Ferrucci LM, Sanft T, Irwin ML, et al. Changes in diet quality in a randomized weight loss trial in breast cancer survivors: the lifestyle, exercise, and nutrition (LEAN) study. NPJ Breast Cancer. 2016;2:16026. 28721384 10.1038/npjbcancer.2016.26 PMC5515327

[40] Voskuil DW, van Nes JG, Junggeburt JM, van dV, van Leeuwen FE, de Haes JC. Maintenance of physical activity and body weight in relation to subsequent quality of life in postmenopausal breast cancer patients. AnnOncol. 2010;21(10):2094–101. 10.1093/annonc/mdq151 20357033

[41] Clamp LD, Hume DJ, Lambert EV, Kroff J. Successful and unsuccessful weight-loss maintainers: strategies to counteract metabolic compensation following weight loss. J Nutr Sci. 2018;7:e20. 29988905 10.1017/jns.2018.11 PMC6033771

[42] Ostendorf DM, Caldwell AE, Creasy SA, Pan Z, Lyden K, Bergouignan A, et al. Physical activity energy expenditure and total daily energy expenditure in successful weight loss maintainers. Obesity (Silver Spring). 2019;27(3):496–504. 30801984 10.1002/oby.22373 PMC6392078

[43] Hauner D, Rack B, Friedl T, Hepp P, Janni W, Hauner H. Rationale and description of a lifestyle intervention programme to achieve moderate weight loss in women with non-metastatic breast cancer: the lifestyle intervention part of the SUCCESS C study. BMJ Nutr Prev Health. 2020;3(2):213–9. 33521531 10.1136/bmjnph-2020-000119 PMC7841841

[44] Villarini A, Pasanisi P, Traina A, Mano MP, Bonanni B, Panico S, et al. Lifestyle and breast cancer recurrences: the DIANA-5 trial. Tumori. 2012;98(1):1–18. 22495696 10.1177/030089161209800101

[45] Ligibel JA, Barry WT, Alfano C, Hershman DL, Irwin M, Neuhouser M, et al. Randomized phase III trial evaluating the role of weight loss in adjuvant treatment of overweight and obese women with early breast cancer (Alliance A011401): study design. NPJ Breast Cancer. 2017;3:37. 28948213 10.1038/s41523-017-0040-8 PMC5608692

[46] van Cappellen-van Maldegem SJM, Mols F, Horevoorts N, de Kruif A, Buffart LM, Schoormans D, et al. Towards OPtimal TIming and Method for promoting sUstained adherence to lifestyle and body weight recommendations in postMenopausal breast cancer survivors (the OPTIMUM-study): protocol for a longitudinal mixed-method study. BMC Womens Health. 2021;21(1):268. 34229690 10.1186/s12905-021-01406-1 PMC8258491

