# Supplementary material for: North of England Women’s Diet and ActivitY - After Breast Cancer (NEWDAY-ABC) intervention in women diagnosed with early oestrogen-positive, HER2-negative breast cancer: a randomised controlled feasibility study
Source: Pilot Feasibility Stud. 2025 Aug 7;11:108. doi: 10.1186/s40814-025-01689-3 (PMC12330054; doi:10.1186/s40814-025-01689-3)
Supplement: Supplementary file 1 — Additional file 1: Supplementary table: Results. [file 40814_2025_1689_MOESM1_ESM.docx]

|  | **Intervention group**  Mean (SD) [Range]  n=7 | | | **Control group**  Mean (SD) [Range]  n=4 | | Mean difference (95% CI) at 6 months adjusting for baseline score |
| --- | --- | --- | --- | --- | --- | --- |
|  | Baseline | | 6 months | Baseline | 6 months |  |
| **EORTC-QLQ-C30**  (Higher number reflects improvement) | | | | | | |
| *Summary score* | 83.9 (11.9)  [61.1-95.9] | 86.1(5.9)  [76.5-93.4] | | 66.0 (19.0)  [41.5-87.8] | 70.8 (18.3)  [49.1-90.6] | 3.08 (-7.5, 13.8) |
| **EORTC-BR23 Breast Module** | | | | | | |
| **Functional Scales (Higher number reflects improvement)** | | | | | | |
| *Body Image* | 60.7 (25.8)  [33.3-83.3] | 79.8 (10.6)  [58.3-91.7] | | 35.4 (10.5)  [25.0-50.0] | 50.0 (39.1)  [16.7-91.7] | 19.2 (-21.0, 59.5) |
| *Sexual Functioning* | 21.4 (12.6)  [0.0-33.3] | 28.6 (12.6)  [0.0-33.3] | | 8.3 (16.7)  [0.0-33.3] | 8.3 (16.7)  [0.0-33.3] | 17.0 (-6.3, 40.4) |
| *Future Perspective* | 57.1 (16.3)  [33.3-66.7] | 66.7 (0.0)  [66.7-66.7] | | 25.0 (31.9)  [0.0-66.7] | 33.3 (38.5)  [0.0-66.7] | 10.3 (-18.7, 39.2) |
| **Symptom Scales (Lower number reflects improvement)** | | | | | | |
| *Systemic Therapy side-effects* | 18.4 (7.5)  [9.5-28.6] | 23.1 (5.8)  [14.3-28.6] | | 26.2 (6.1)  [19.1-33.3] | 29.8 (9.8)  [19.1-42.9] | -3.3 (-15.3, 8.7) |
| *Breast Symptoms* | 15.5 (15.5)  [0.0-33.3] | 9.5 (10.1)  [0.0-25.0] | | 27.1 (21.9)  [8.3-58.3] | 27.1 (20.8)  [16.7-58.3] | -10.1 (-24.5, 4.3) |
| *Arm Symptoms* | 12.7 (10.0)  [0.0-22.2] | 11.1 (11.1)  [0.0-33.3] | | 30.6 (19.0)  [11.1-55.6] | 41.7 (14.0)  [22.2-55.6] | -29.6 (-52.2, -6.9) |
| **EQ-5D**  (Higher number reflects improvement) | | | | | | |
| *Utility score* | 0.79 (0.08)  [0.63-0.88] | 0.82 (0.11)  [0.66-1.00] | | 0.58 (0.33)  [0.10-0.84] | 0.63 (0.31)  [0.25-1.00] | 0.02 (-0.18, 0.22) |
| *Visual Analogue Scale* | 75.0 (13.5)  [50-90] | 85.0 (7.1)  [75-95] | | 56.3 (20.6)  [30-75] | 71.3 (18.4)  [50-95] | 7.1 (-12.1, 26.2) |
| **Fear of Cancer Recurrence**  (Lower number reflects improvement) | | | | | | |
| Overall scale | 22.7 (6.3)  [17-35] | 19.7 (3.1)  [14-23] | | 30.8 (11.1)  [15-39] | 30.5 (13.2)  [11-40] | -4.4 (-12.4, 3.7) |
| **FACIT Fatigue Scale**  (Higher number reflects improvement) | | | | | |  |
| Overall score | 16.4 (8.1)  [8-27] | 13.3 (5.5)  [7-22] | | 24.3 (12.0)  [13-37] | 24.5 (9.6)  [12-32] | -6.1 (-11.8,-0.4) |
| **Body Image Scale**  (Lower number reflects improvement) | | | | | |  |
| Overall score | 10.6 (6.2)  [3-20] | 6.0 (4.1)  [0-13] | | 16.8 (6.9)  [11-25] | 18.0 (10.0)  [7-27] | -6.7 (-13.2, -0.3) |
